# Association between intestinal microbiome and inflammatory bowel disease: Insights from bibliometric analysis

**DOI:** 10.1016/j.csbj.2022.04.006

**Published:** 2022-04-07

**Authors:** Pengfei Xu, Tengteng Lv, Shenghui Dong, Zhihao Cui, Xinyuan Luo, Baolei Jia, Che Ok Jeon, Jie Zhang

**Affiliations:** a School of Bioengineering, State Key Laboratory of Biobased Material and Green Papermaking, Qilu University of Technology (Shandong Academy of Sciences), Jinan 250353, China; b Department of Life Science, Chung-Ang University, Seoul 06974, Republic of Korea

**Keywords:** IBD, inflammatory bowel disease, CD, crohn's disease, UC, ulcerative colitis, WoSCC, Web of Science Core Collection, TNF, tumor necrosis factor, SCFA, short-chain fatty acids, IgA, Immunoglobulin A, PRR, pattern recognition receptors, TLR, toll-like receptors, NLR, nod-like receptors, CLR, C-type lectin receptors, IL, interleukin, Bibliometrics, Metagenomics, Crohn's disease, ulcerative colitis, Probiotics

## Abstract

The gut microbiome is highly linked to inflammatory bowel disease (IBD). A total of 3890 publications related to the two terms from 2000 to 2020 were extracted from the Web of Science Core Collection to study the association from a bibliometric perspective. Publications on this topic have grown rapidly since 2008. The United States and Harvard University are the country and institution with the largest number of publications, respectively. Inflammatory Bowel Diseases is the most productive journal with 211 published articles. The most influential journal in this field is Gut with 13,359 citations. The co-citation analysis of references showed that the IBD-related topics with the highest focus are “gut microbiota,” “metagenomics,” “bacterial community,” “fecal microbiota transplantation,” “probiotics,” and “colitis-associated colorectal cancer.” Keyword cluster and keyword burst analyses showed that “gut microbiota,” “metagenomics,” and “fecal microbiota transplantation” are currently the most researched topics in the field of IBD. The literature in this field is mainly distributed between alterations of the intestinal microbiota, microbial metabolites, and related host signaling pathways. Probiotic treatment also frequently appears in literature. This bibliometric analysis can guide future research and promote the development of the field of gut microbiome and IBD.

## Highlights

• Intestinal microbiota has been increasingly studied in the field of IBD over the last 20 years.
• The gut microbiome, metabolites, and their corresponding host signaling pathways are highly associated with IBD.
• Probiotics may relieve IBD as a complementary therapy.
• The pathogenesis and treatment strategies of IBD need to be further studied.

## Graphical abstract

**Figure:**
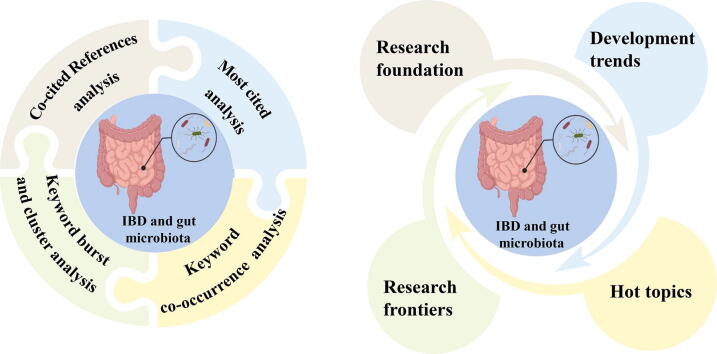

## 1 Introduction

A large variety of bacteria, viruses, and fungi are present in the intestines, which provide a natural habitat for these microorganisms that are collectively referred to as the intestinal microbiota [1], [2], [3]. This microbiota relies on the host’s intestine to live, has metabolic functions that the host does not, and facilitates several physiological functions of the host [4], [5]. Additionally, gut microbiota plays an important role in the metabolism, development, and immune system of the host [6].

Inflammatory bowel disease (IBD), including Crohn's disease (CD) and ulcerative colitis (UC), is a chronic intestinal immune-inflammatory disease of unknown etiology [7], [8]. IBD is characterized by a loss of tolerance to intestinal microorganisms and abnormal host intestinal immunity, leading to intestinal mucosal inflammation [9]. The clinical manifestations are diarrhea, abdominal pain, and bloody stools. Recent studies have shown that this disease may be related to intestinal microbial imbalance or autoimmune factors [10], [11]. With the development of technologies such as metagenomics and metatranscriptomics, the physiological functions of the gut microbiota have gradually been discovered and confirmed at the genetic level [12], [13]. Differences in gut microbiomes between healthy individuals and individuals with IBD have been widely studied [14]. Patients with IBD are reported to have a decreased diversity of the intestinal microbiota, an increase in harmful metabolites, and an altered host signal pathway with an increase in the content of pro-inflammatory factors [15], [16], [17].

In the past two decades, human and animal studies on the relationship between the gut microbiome and IBD have increased substantially. However, the relationship between the gut microbiome and IBD has not been systematically assessed through bibliometric analysis. In this study, we used bibliometric analysis to fill gaps in the literature. Bibliometrics takes the literature system and bibliometric characteristics as the research object and uses quantitative research methods to analyze the distribution, relationship, change in literature, and progress in a certain field [18], [19], [20], [21]. In this study, it was used to analyze the cooperation, literature influence, journals, researchers, references, and keywords in the field of the gut microbiome and IBD from 2000 to 2020. The aim of this study was to reveal the current status of the association between IBD and gut microbiome [22].

## 2 Methods

### 2.1 Data retrieval

Literature related to the intestinal microbiome and IBD from 2000 to 2020 was searched using the Web of Science Core Collection (WoSCC) database. The English language was used to search the subject field (including title, author keywords, abstract), and the Boolean operators were set to TS= (gastrointestinal OR gut OR intestinal OR bowel) AND (microbiome OR microbiota OR microorganism OR microbe) AND (“inflammatory bowel disease”) (Search deadline: July 26, 2021). The operator AND can search for records containing all search terms separated by this operator, whereas the operator OR can search for records containing any search terms separated by this operator.

### 2.2 Data analysis

Citespace [23] (version 5.8.R1.7z) and VOSviewer [24] (version 1.6.15) software were used to perform reference co-citation, author timeline, cluster, and keyword burst analyses. The Bibexcel [25] (https://homepage.univie.ac.at/juan.gorraiz/bibexcel/) software was used to analyze the volume of publications, cooperation relationships, and keyword co-occurrence relationships between countries or institutions. The Gephi [26] (v.0.9.2; https://gephi.org/) software was used to construct social network graphs of countries, institutions, and keywords for data visualization.

## 3 Results

### 3.1 Publication amount analysis shows that the microbiome studies in IBD increased markedly

**Fig. 1:**
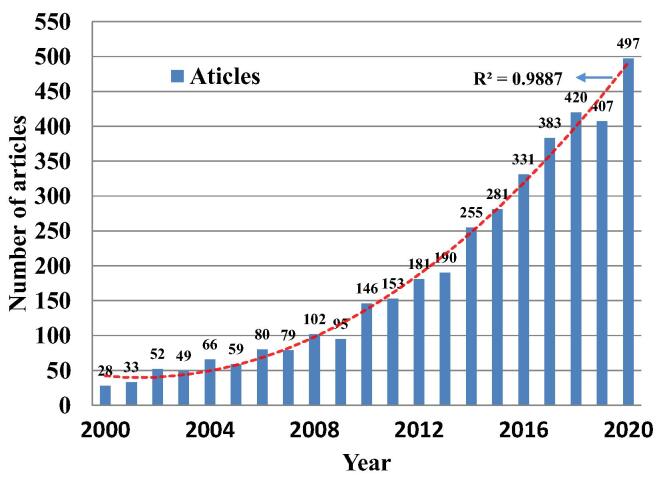
Annual trend and polynomial fitting curve of published articles in the field of gut microbiome and IBD in the database of WoSCC from 2000 to 2020.

To analyze the development trend in the field of IBD and intestinal microbiome, related publications were retrieved from WoSCC, the world's largest multi-disciplinary and comprehensive citation index database. A preliminary search of 7117 articles was conducted for publications from 2000 to 2020. Among the publication types, “Article” had 4326 records, accounting for 60.78% (Table 1). The “Articles” can help evaluate the trends and research hotspots of related fields to the greatest extent, given their prevalence. Therefore, we focused on analyzing these articles in subsequent research. After the artificial screening, the “Articles” that were irrelevant or less relevant to the research content were excluded. In total, 3890 articles were included in this study. The total number of articles on the gut microbiome and IBD showed an increasing trend (Fig. 1). During 2000–2008, there was little research in this field, and the number of articles increased slowly. From 2008 to 2020, the number of journal articles published in this field increased markedly, with slight fluctuations during this period. For 2000–2020, the annual growth rate was calculated as: (2020 value/2000 value) × (1/20) = 15.5%.

**Table 1:** Types of documents published from 2000 to 2020.

Documents type	NR	Percentage (%)
Article	4326	60.78
Review	2366	33.24
Meeting abstract	181	2.54
Proceedings paper	180	2.53
Editorial material	138	1.94
Book chapter	85	1.19
Letter	39	0.55
Correction	10	0.14
Data paper	7	0.10
Early access	6	0.08
News item	6	0.08
Retracted publication	4	0.06
Biographical item	1	0.01

NR: number of published documents.

### 3.2 Publication countries, institutions, and authors analysis show that the USA is in the leading position in the field

Countries and institutions analyses showed that the 3890 articles were from 85 countries/regions (Fig. S1). The majority of publications (1414) were from the US, accounting for 36.3% of the total, followed by China (562) and the United Kingdom (364), accounting for 13.7% and 9.4% of the total, respectively. More than 152 research institutions have contributed to IBD and gut microbiome research. Harvard University had the largest number of publications (101), followed by Massachusetts General Hospital (84) and the University of Toronto (61).

**Fig. 2:**
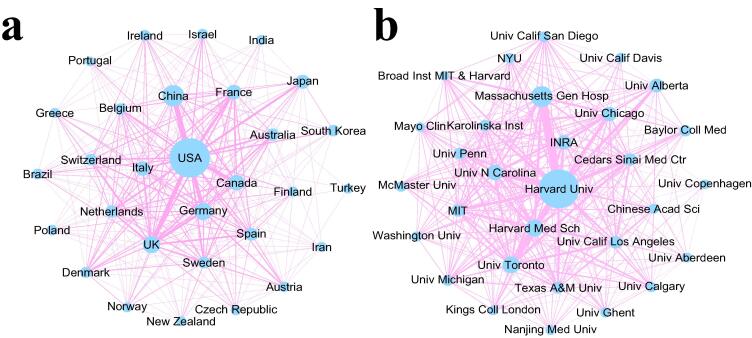
The cooperation network of the top 30 most productive countries and institutions. The size of the circle indicates how often a country or institution publishes documents, and the line width between two points represents the level of cooperation between countries or institutions.

Effective international cooperation plays an important role in promoting academic exchanges and cooperation. Fig. 2a shows the top 30 cooperative studies with the largest number of publications in the field from 2000 to 2020. Among them, the United States and China are the two major producers of research in this field, and cooperation between them far exceeds that of other countries. Second, cooperation between the United States and the United Kingdom, Canada, Germany, and other countries is relatively frequent. Although there is close cooperation among the 30 countries, they are mainly from countries with high productivity, such as the United States, China, Canada, and Germany. Furthermore, the partnerships between institutes were also analyzed (Fig. 2b), which showed that Harvard University maintains close partnerships with Massachusetts General Hospital and the University of Toronto.

Up to 2020, 20,764 authors have participated in the publication of the 3890 articles. Detailed information on the 10 most prolific authors is provided in Table S1. Ramnik J. Xavier is the most prolific author in the field of IBD and gut microbiome, followed by Harry Sokol. Ramnik J Xavier is the most influential author with the highest number of citations (11506) and the highest H-index (34).

Fig. S2 shows the author's timeline analysis based on keywords that can present the research directions of active researchers in this field and their results over time (Fig. S2). The data showed that the research fields of active researchers are mainly distributed in six clusters: dendritic cells, dysbiosis, metabolomics, translational research, CD, and intestinal inflammation.

### 3.3 Journal analysis indicated the paper are published in both professional and comprehensive journals

Journal analysis has been widely used to study the interdisciplinary structure of a given academic field. A journal with the most publications in a certain field can provide suggestions for the communication and dissemination of discoveries. From 2000 to 2020, a total of 874 journals worldwide published papers on the gut microbiome and IBD. The top 20 journals with the highest number of articles accounted for 33.70% of the total number of articles (Table S2). Inflammatory bowel diseases is the most productive journal with a total of 211 articles. This was followed by PLoS One (465) and Gut (415). Although Gastroenterology ranks 9th in terms of the number of articles published, it has the highest IF among the 20 journals (22.682), followed by Gut (22.059). Gut is the most-cited journal with 13,359 citations, followed by Proceedings of the National Academy of Sciences of the United States of America (13289) and Gastroenterology (12851).

### 3.4 Analysis of co-cited references reveals the research foundation in the field

Co-cited references are those that are often cited together with other articles and can be regarded as the basis of research in a particular field. Based on the log-likelihood ratio algorithm in the Citespace software, the six largest clusters of the reference co-citation network were: “gut microbiota (Cluster #0),” “metagenomics (Cluster #1),” “bacterial community (Cluster #2),” “fecal microbiota transplantation (Cluster #5),” “probiotics (Cluster #7),” and “colitis-associated colorectal cancer (Cluster # 8)” (Fig. S3). All clusters were constructed based on the keywords extracted from the references. The total modularity Q value was 0.6717, and the average contour of each cluster was greater than 0.7, indicating that the cluster structure was significant and that the results were highly reliable [27].

Burst detection can be used to identify articles that have attracted the attention of researchers. Based on the analysis of the 3890 original articles, we found that 8 showed the characteristics of citation bursts between 2000 and 2020 (Fig. S4). These articles covered the period from 2000 to 2020. As the burst time of the citation could explain the research hotspots, the data from these articles have promoted the output of research and articles in the field of intestinal microbiome and IBD.

### 3.5 Most cited analysis elucidate the most influential paper in the field

The number of citations can also reflect the hotspots and trends in research. The results showed that the most cited article is “Host-microbe interactions have shaped the genetic architecture of inflammatory bowel disease” written by Jostins et al., which was published in Nature in 2012, and the number of citations was 2632 (Table 2). This was followed by a paper titled “Molecular-phylogenetic characterization of microbial community imbalances in human inflammatory bowel diseases” published in “Proceedings of the National Academy of Sciences of the United States of America” in 2007.

**Table 2:** The most-cited paper from 2000 to 2020 in the field of IBD and gut microbiome.

Title	Year	TC	Journal	First author
Host-microbe interactions have shaped the genetic architecture of inflammatory bowel disease	2012	2710	Nature	Luke Jostins
Molecular-phylogenetic characterization of microbial community imbalances in human inflammatorybowel diseases	2007	2599	Proc Natl Acad Sci U S A	Daniel N. Frank
Faecalibacterium prausnitzii is an anti-inflammatory commensal bacterium identified by gutmicrobiota analysis of Crohn disease patients	2008	2275	Proc Natl Acad Sci U S A	Harry Sokol
A microbial symbiosis factor prevents intestinal inflammatory disease	2008	1439	Nature	Sarkis K. Mazmanian
Dysfunction of the intestinal microbiome in inflammatory bowel disease and treatment	2012	1359	Genome Biology	Xochitl C. Morgan
Genome-wide association study identifies new susceptibility loci for Crohn disease and implicates autophagy in disease pathogenesis	2007	1341	Nature Genetics	John D. Rioux
Inducible Foxp(3+) regulatory T-cell development by a commensal bacterium of the intestinal microbiota	2010	1231	Proc Natl Acad Sci U S A	June L. Round
Specific Microbiota Direct the Differentiation of IL-17-Producing T-Helper Cells in the Mucosa of the Small Intestine	2008	1101	Cell Host & Microbe	Ivaylo I. Ivanov
Intestinal Inflammation Targets Cancer-Inducing Activity of the Microbiota	2012	1076	Science	Janelle C. Arthur
Mucosal microbiota in inflammatory bowel disease	2002	994	Gastroenterology	Alexander Swidsinski

TC: total citations.

### 3.6 Keyword co-occurrence analysis reveals the hot topics in the field

**Fig. 3:**
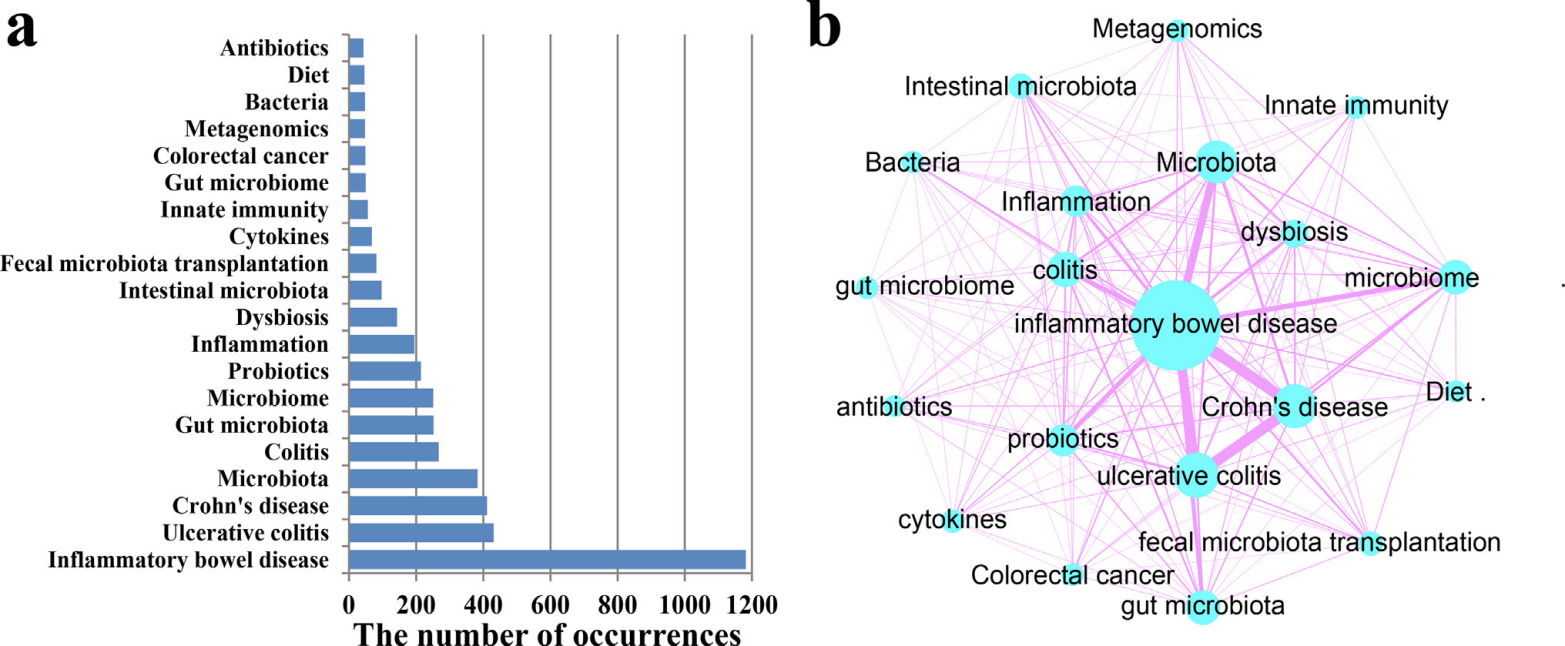
The top 20 keywords with the highest frequency. (a) Frequency of keyword occurrence; (b) Keyword co-occurrence network diagram. The size of the circle indicates the frequency of keyword occurrence, and the width of the line indicates the frequency of keyword co-occurrence.

Keyword co-occurrence analysis is designed to study the co-occurrence relationship between keywords in a set of publications that can reflect popular topics. This section showed the co-occurrence relationship of the top 20 keywords with the highest frequencies. The term “inflammatory bowel disease” with 1182 occurrences ranked first, followed by “ulcerative colitis” with 431 occurrences and “Crohn's disease” 411 occurrences (Fig. 3a). Additionally, the keywords “dysbiosis,” “fecal microbiota transplantation,” “cytokines,” “innate immunity,” and “diet” were also listed in the top 20 keywords. Fig. 3b shows the top 20 keyword co-occurrence networks. CD had the highest correlation with UC with 218 co-occurrences. The co-occurrences of IBD and UC, CD, and microbiota were 177, 172, and 117, respectively.

### 3.7 Keyword burst analysis and cluster analysis reveals the research frontiers in the field

**Fig. 4:**
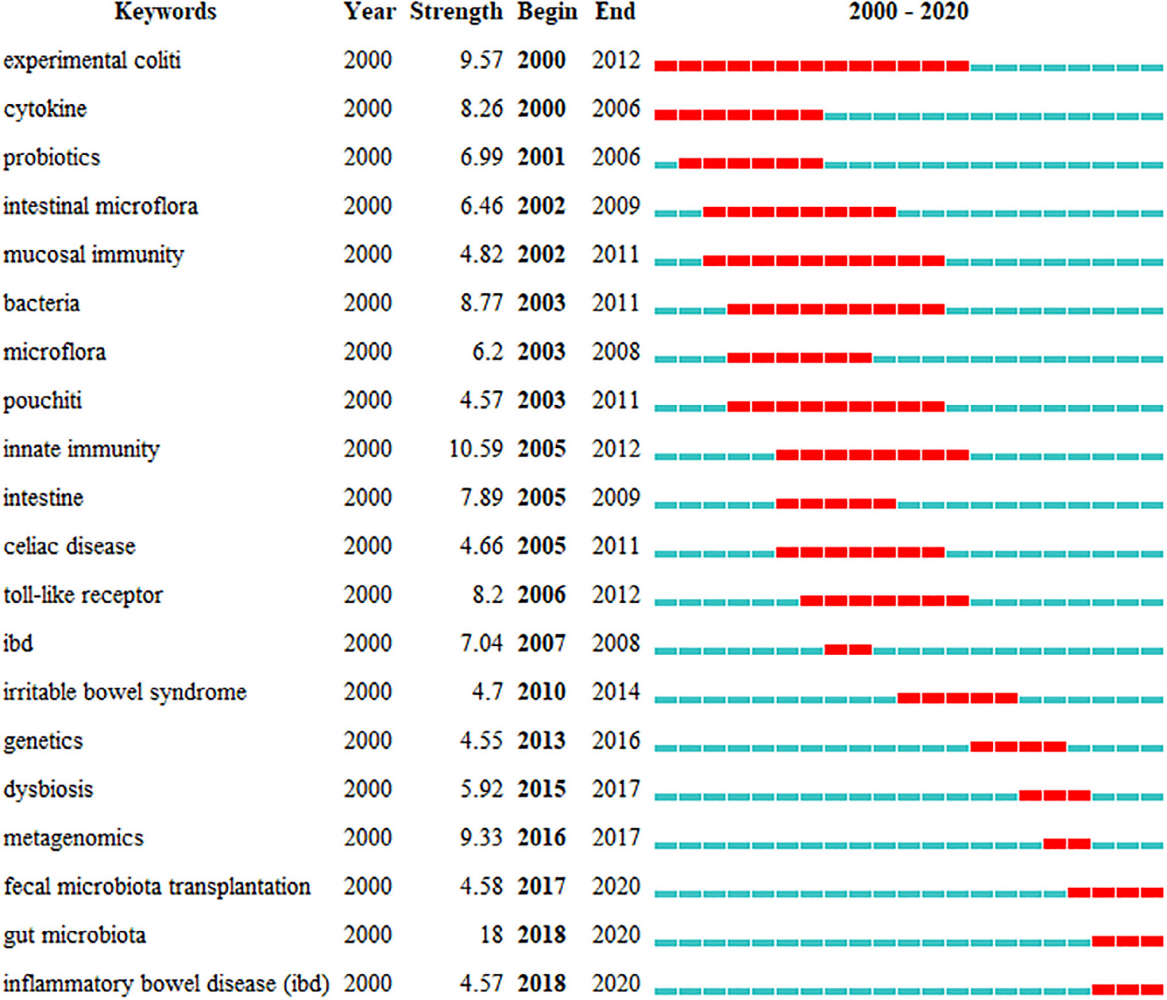
Top 20 keywords with the strongest citation bursts. The blue bar represents the time period when the keyword appears; the red bar represents the time period when the keyword bursts.

“Author keywords” in the WoSCC database can be regarded as an emerging topic in this field. Considering that the analysis results of different bibliometric tools may be different, we used the keyword clustering map of VOSviewer and keyword burst analysis in the Citespace software to conduct an overlap analysis to determine the research frontiers in this field. The burst analysis of keywords involves two attributes (burst intensity and duration) which can reveal the sudden changes of keywords in a specific period and can be used as an indicator of emerging research directions [27]. Among the top 20 keywords with the highest burst intensity, we were particularly interested in keywords that had research significance. These keywords indicated the research trends in the field of IBD and gut microbiome (Fig. 4). From 2000 to 2020, the burst intensity of “gut microbiota” was the highest (18), followed by those of “innate immunity” (10.59) and “experimental colitis” (9.57). The top 20 keyword outbreak cycles with the highest occurrences covered the entire period from 2000 to 2020. “Experimental colitis” and “cytokine” have been research hotspots since 2000, and the duration of “Experimental colitis” outbreaks is the longest (2000–2012), followed by “probiotics” and “intestinal microbiota,” which gained popularity in 2001 and 2002, respectively. “Innate immunity” was in focus during 2005–2012, followed by “toll-like receptor,” which marked the entry of a new era in microbiome research. Gut microbiota and IBD became outbreak keywords in 2018. Although the burst period of “gut microbiota” is relatively short (2018–2020), the burst intensity is the highest. It can be seen that “gut microbiota” is a widely popular topic in current research related to IBD.

**Fig. 5:**
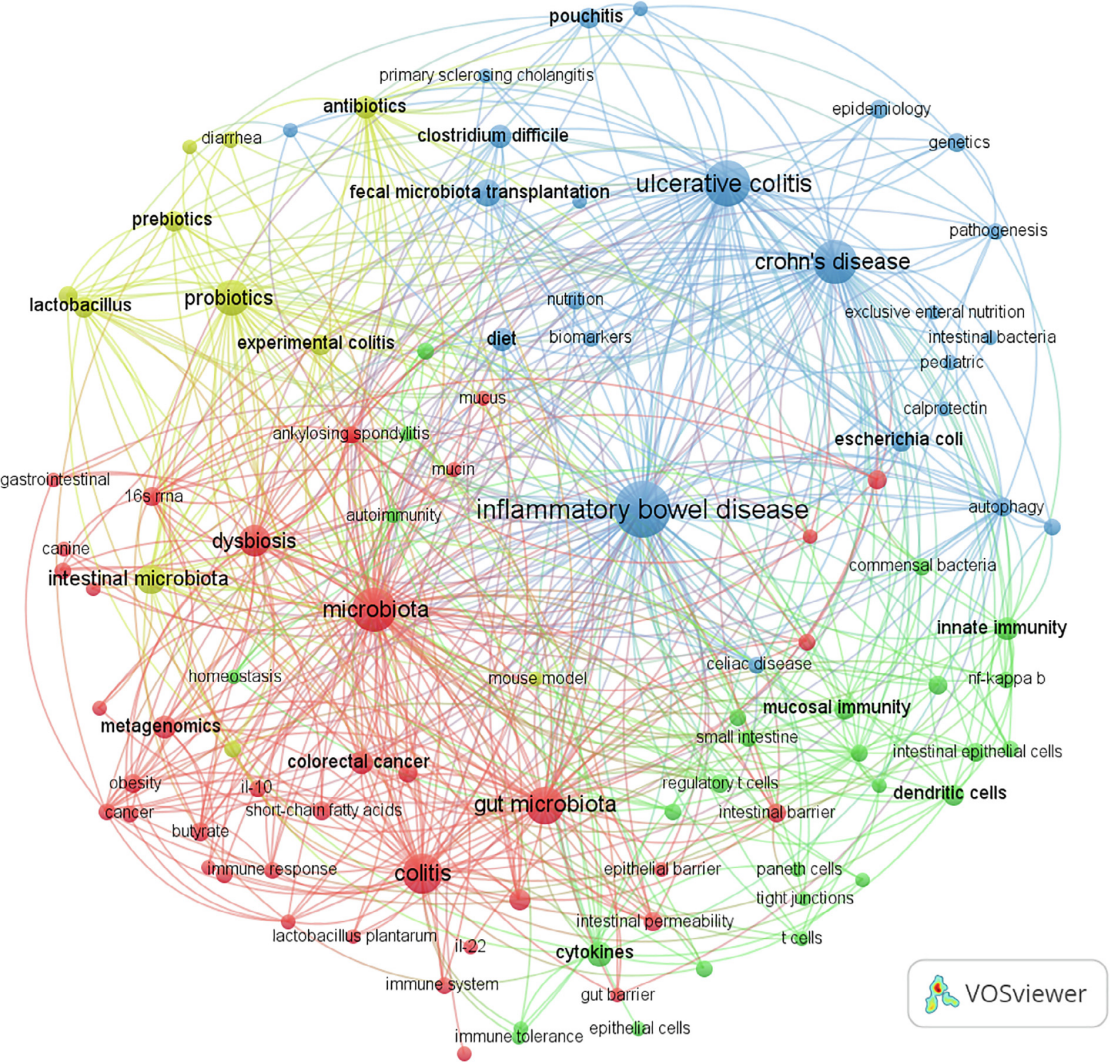
Author keyword cluster analysis. The size of the circle indicates the frequency of keyword occurrence; the color of the circle indicates the type of clustering: changes in intestinal flora in IBD (blue), changes in metabolites (red), changes in host signaling pathways caused by flora and metabolites (green), probiotic treatment (yellow); the connection indicates the co-occurrence relationship of keywords. The most relevant nodes belong to the same cluster with the same color, which indicates a close cooperative relationship. The size of the node and the width of the connecting line are related to the relative degree of co-occurrence between nodes and the relative strength of the co-occurrence relationship.

Fig. 5 shows the co-occurrence network of the 101 keywords with more than 10 occurrences. Four different clusters, including intestinal microbiota in IBD, microbial metabolites, host signaling pathways caused by microbiota and metabolites, and treatment for IBD were established using the “author keywords” parameter and the “Louvain” clustering algorithm. The most relevant nodes belonged to the same cluster with the same color, indicating a close cooperative relationship. The size of the node and the width of the connecting line are related to the relative degree of co-occurrence between the nodes and the relative strength of the co-occurrence relationship.

## 4 Discussion

To date, studies have shown that the intestinal microbiome is associated with a variety of digestive tract diseases, particularly IBD. The role of changes in intestinal microbes in the pathogenesis of IBD has attracted increasing attention, making intestinal microbes a research hotspot in the field of IBD in the past two decades. Currently, bibliometric analysis is increasingly being used to examine the status and trends of specific fields [28], [29]. However, to date, no bibliometric analysis has been conducted in the field of IBD, particularly on the research of intestinal microbes in this field. Next, we combined the frequency of the occurrence of keywords in each category and the co-occurrence relationship to present an in-depth analysis of the literature under each category.

Research trends in the field from literature analysis. In the reference cluster analysis, the largest occurrence of Cluster #0 was “gut microbiota” with the average year of 2015. The next four largest clusters were “metagenomics” (average year 2011), “bacterial community” (average year 1998), “fecal microbiota transplantation” (average year 2014), “probiotics” (average year 2002). The clustering timeline shows the development of the intestinal microbiome from 2000 to 2020. The average year of “gut microbiota” was 2015, suggesting that the research on gut microbiota is still in its early stages. It is worth noting that “metagenomics” plays a decisive role in the study of the gut microbiome. Before “metagenomics,” the research was limited to single bacteria or probiotics, and metagenomics could be combined with the whole gut microbiome through shotgun sequencing.

### 4.1 Analysis of research hotspots from the high-citation literature

**Fig. 6:**
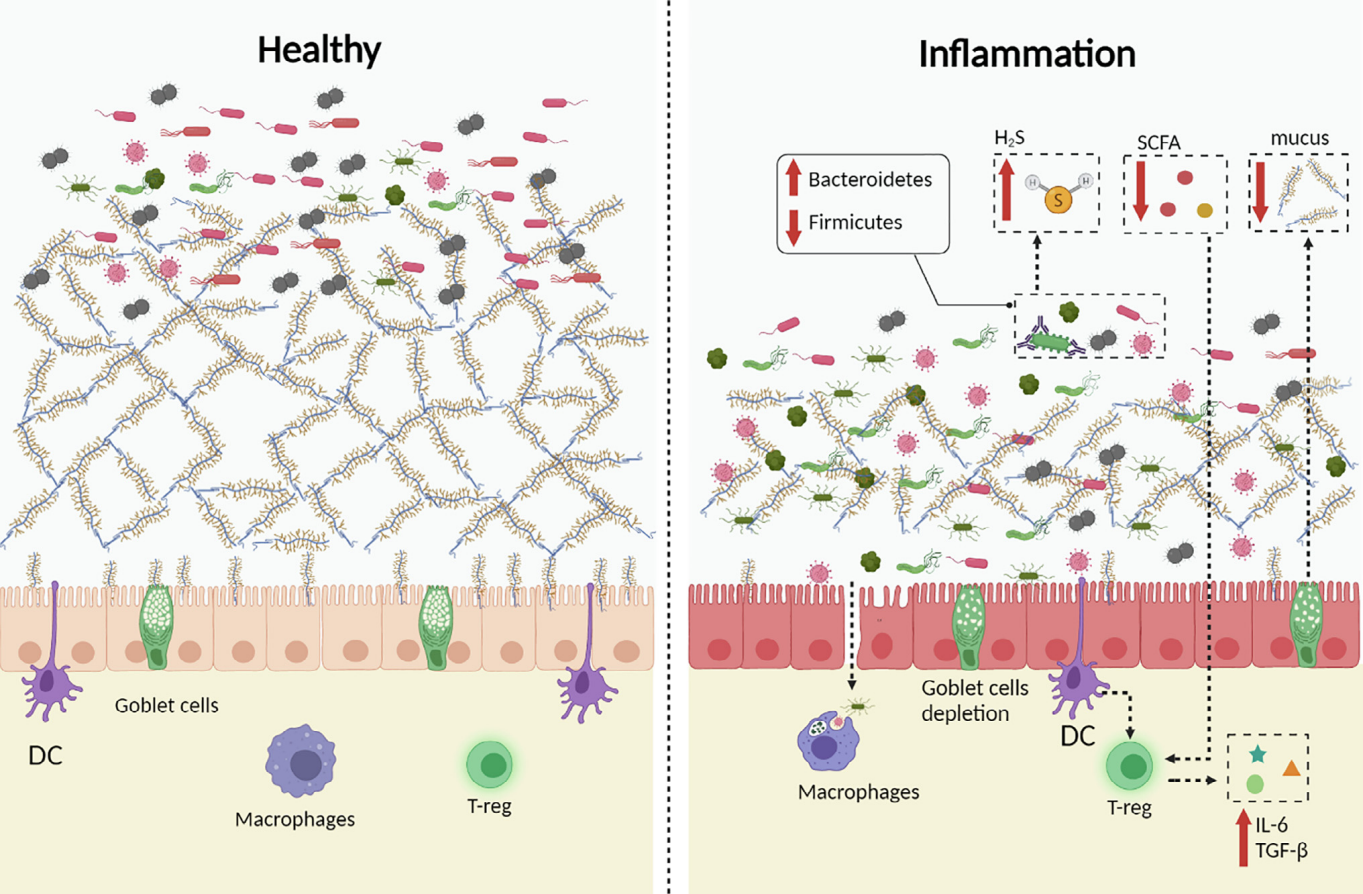
The alterations of intestinal microbiome in IBD. In patients with IBD, the community of the intestinal microbiota is altered as indicated in figure. Furthermore, the abundance of microbial metabolites, such as H_2_S and short-chain fatty acids are changed. In IBD, gut microbiota could also impact the function of macrophages and dendritic cells (DC). Additionally, the microbiota affects the production of pro-inflammatory factors such as TNF-α and interleukin (IL)-6. Finally, the mucin layer becomes thinner in IBD because of impaired goblet cell function.

Analysis of the top 10 most-cited articles showed that studies have focused on three subjects: 1) alteration of the gut microbiome in IBD, 2) alteration of microbial metabolites in IBD, and 3) relationship between host genetics and gut microbiome in IBD. First, metagenomic analysis revealed a notable decrease in members of Firmicutes and increase in members of Bacteroidetes, as reported previously [30], [31], [32]. In addition, this alteration can promote tumorigenesis by inducing the proliferation of microorganisms with genotoxic capabilities [33]. Second, microbial metabolites have an important relationship with IBD [34]. For example, polysaccharide A secreted by *Bacteroides fragilis* mediates the transformation of CD4 cells into Foxp3 T cells, which produce inducible IL-10, mediate mucosal surface tolerance, and prevent intestinal inflammation [35], [36]. *Faecalibacterium prausnitzii* exerts anti-inflammatory effects by secreting metabolites that block NF-κB activation and IL-8 secretion [37]. Third, multiple IBD susceptibility loci were discovered using meta-analysis of genome-wide association studies. These loci have also been implicated in other immune-mediated diseases, most notably ankylosing spondylitis and psoriasis [38], [39]. In the next sections, we discuss the related alterations in the gut microbiome and their metabolites (Fig. 6).

### 4.2 Alteration of the intestinal microbiota in IBD using keyword analysis

The development of metagenomic sequencing has shown that IBD is strongly correlated with gut microbiota. Several studies on intestinal or fecal microbiota have found that the composition and metabolism of the gut microbiota are significantly different in IBD patients compared to those in normal populations. The most consistent change observed in most patients with IBD is the decrease in the diversity of the gut microbiota [30], [40], [41]. In the intestinal mucosal microbiota, certain *Firmicutes* members often decrease in abundance [14], [42], [43] (Fig. 6). Among the members, the butyrate-producing bacterium *F. prausnitzii* has been repeatedly observed to be significantly decreased in IBD patients [44], [45]. In contrast, in the stool samples of patients with IBD, the content of pathogenic bacteria, such as *Enterobacteriaceae*, has increased [46], [47]. In *Enterobacteriaceae*, adherent-invasive *E. coli* is usually found in the intestinal mucosa of patients with IBD, particularly in those with ileal mucosal lesions. *E. coli* also invades host cells [48]. In addition, the adherent-invasive *E. coli* can survive and replicate in host cells without triggering host cell death, and simultaneously release a large amount of tumor necrosis factor (TNF)-α [49]. Compared to the healthy group, the level of *Fusobacterium* in the feces of IBD patients was significantly increased [50], [51], [52]. *Fusobacterium* isolated from IBD patients is more aggressive than that isolated from normal tissues [53], [54]. However, the presence and abundance of specific bacterial species vary with disease severity and sampling site (feces and colon) [55]. In conclusion, the diversity of the intestinal microbiota of patients with IBD is reduced and the abundance of certain microbes changes. However, there is no evidence that changes in intestinal microbes are the cause or result of IBD [56].

### 4.3 Changes in microbial metabolites during IBD

Studies have shown that microbial metabolites are involved in IBD [57], [58], [59] (Fig. 6). For example, the production of hydrogen sulfide by sulfate-reducing bacteria increased in the inflamation conditions, which may have therapeutic or toxic effects, depending on its concentration [17], [60], [61]. Metabolomic analysis revealed that the levels of short chain fatty acids (SCFAs), methylamine, and trimethylamine in IBD patients were significantly reduced [62]. The reduction in the levels of SCFAs can have negative consequences as the molecules help maintain the integrity of the epithelium, thereby protecting the host from bacterial invasion and infection. For example, butyric acid can inhibit pro-inflammatory cytokine signaling pathways. Butyric acid-producing bacteria and their culture supernatants improved intestinal inflammation in animal models of colitis [37]. Additionally, a few studies have confirmed that the levels of some butyric acid-producing bacteria (such as *Clostridium butyricum and Bifidobacterium*) are significantly lower in patients with IBD [63]. In contrast, butyrate-mediated G protein-coupled receptor activation improves intestinal mucosal barrier function by inhibiting histone deacetylase, regulating intestinal macrophages, and regulating T cells [64]. Immunoglobulin A (IgA) maintains homeostasis in the intestinal mucosa and is the most abundant immunoglobulin in the mammalian gut. According to reports, SCFAs are involved in the production of IgA, which has been confirmed to be positively correlated with SCFAs [65], [66]. Acetic acid is an SCFA and a major gut microbial metabolite. Acetic acid not only increases the production of IgA in the colon but also changes the ability of IgA to bind to specific microorganisms (including *Enterobacter*) and regulate IgA production to maintain intestinal mucosal homeostasis [67].

### 4.4 Host signaling pathway changes associated to microbiota

The human immune system is generally tolerant to symbiotic microbiota (Fig. 6). However, microbial dysbiosis stimulates the immune system of individuals susceptible to IBD, which may be the underlying pathogenesis of IBD [68]. Pattern recognition receptors (PRR) in intestinal mucosal epithelial cells, including toll-like receptors (TLR), nod-like receptors (NLR), and C-type lectin receptors (CLR) can recognize microbial molecules and trigger and maintain immunity in the intestinal mucosa [69], [70], [71], [72]. The PRR-mediated intestinal cellular immune response includes microbial binding, phagocytosis, antibacterial effects, and cytokine production [73]. In addition, most genetic susceptibility sites related to IBD are associated with PRR. The microbiota further promotes signaling of the inflammasome pathway by inducing the transcription of pro-inflammatory cytokines, such as TNF-α and interleukin (IL)-6 [74], [75], [76]. In addition, the intestinal microbiota regulates the functions of macrophages and dendritic cells to protect the host from pathogen infection and maintain immune tolerance to the intestinal microbes themselves [77]. Finally, IBD is associated with impaired goblet cell function and dysregulated mucins synthesis, leading to a thinner mucin layer and barrier dysfunction [50], [78]. However, the relationship between IBD and mucin needs to be studied further because of the uncertainty in existing research [79].

### 4.5 Probiotic treatment for IBD based on literature analysis

“Probiotic” occurred as a frequent keyword. We further summarized the function of probiotics in the treatment of IBD. Zhang et al. showed that *Lactobacillus plantarum* significantly inhibits the production of intrinsic proinflammatory cytokines during the development of colitis in mice and improves intestinal stability, suggesting that this strain may be effective in controlling colitis symptoms and has therapeutic potential for IBD [80]. Yeo et al. found that *Lactobacillus rhamnosus* strain LDTM 7511 attenuated inflammation and normalized bacterial dysbiosis in the inflamed gut of mice, suggesting that the probiotic has potential as an adjunctive treatment for IBD [81]. Bejarnason et al. investigated the effects of probiotics on adult patients with UC and CD in remission or with mild symptoms. They used a combination of probiotics, including *L. plantarum*, *L. rhamnosus*, *Lactobacillus acidophilus*, and *Streptococcus faecalis*, and found no difference in clinical symptoms after probiotic treatment [84]. Another study also showed that *Lactobacillus GG*, used as a supplement in standard medical therapy, could be ineffective in preventing relapse [85]. Research suggests that there are currently insufficient data on using probiotics for the treatment or relief of IBD, and the application of probiotics on human IBD treatment requires further evaluation.

## 5 Conclusion

We used bibliometrics to analyze the characteristics of publications in the field of IBD and gut microbiome. Generally, the number of publications in this field is increasing. With the help of literature analysis software such as CiteSpace and VOSviewer, we conducted cluster analysis, co-occurrence analysis, and emergent analysis of the literature, and provided research hotspots and development trends in this field during the past two decades. Through our analysis, we found that the gut microbiota plays an important role in IBD, which is a research hotspot in this field and is expected to continue to receive attention in the future. Recently, metagenomics has been widely used to study intestinal microbes. This method can comprehensively and efficiently display the changes in the entire microbiota. Further studies on the mechanisms linking microbiota and IBD will promote IBD therapy.

## CRediT authorship contribution statement

**Pengfei Xu:** Conceptualization, Investigation, Writing – original draft, Visualization, Data curation, Formal analysis. **Tengteng Lv:** Visualization, Data curation. **Shenghui Dong:** Formal analysis. **Zhihao Cui:** Investigation. **Xinyuan Luo:** Visualization. **Baolei Jia:** Funding acquisition, Methodology, Writing – review & editing, Project administration, Methodology, Formal analysis, Writing – review & editing. **Che Ok Jeon:** . **Jie Zhang:** .

## Declaration of Competing Interest

The authors declare that they have no known competing financial interests or personal relationships that could have appeared to influence the work reported in this paper.
