# Supplementary material for: Association between intestinal microbiome and inflammatory bowel disease: Insights from bibliometric analysis
Source: Comput Struct Biotechnol J. 2022 Apr 7;20:1716–25. doi: 10.1016/j.csbj.2022.04.006 (PMC9019919; doi:10.1016/j.csbj.2022.04.006)
Supplement: Supplementary data 1 [file mmc1.pdf]

Supplementary Material

Table S1. The 10 authors who published the most articles from 2000 to 2020 in the field of IBD and gut microbiome.

Author	Organization (Country/Region)	TP	TC	H-index
Ramnik J Xavier	Harvard Med Sch (USA)	41	11506	34
Harry Sokol	Sorbonne Universite (France)	30	7165	26
Scot E Dowd	Mol Res LP MR DNA(USA)	17	1366	15
Jan S Suchodolski	Texas A&M University System(USA)	17	1701	18
Curtis Huttenhower	Harvard University (USA)	17	4416	20
Gerhard Rogler	Univ Hosp Zurich (Switzerland)	15	680	13
Akira Andoh	Shiga Univ Med Sci (Japan)	15	1480	19
Charles N Bernstein	Univ Manitoba (Canada)	14	1331	15
Ashwin N Ananthakrishnan	Massachusetts Gen Hosp (USA)	14	4152	14
Marteau P	Sorbonne Universite (France)	13	4836	18

TP: total publications; TC: total citations.

Table S2. The top journals from 2000 to 2020 in the field of IBD and gut microbiome.

Journal	TP	Percentage (%)	TC	IF (2020)
Inflamm. Bowel Dis	211	5.42	11748	5.325
PLoS One	147	3.78	7695	3.24
Gut	90	2.31	13359	22.059
World J. Gastroenterol	88	2.26	5886	5.742
Sci Rep	66	1.70	2034	4.379
Dig. Dis	65	1.67	1639	2.404
J. Crohns Colitis	62	1.59	1812	9.071
Front. Microbiol	58	1.49	1713	5.64
Gastroenterology	56	1.44	12851	22.682
Gut Microbes	56	1.44	1583	10.245
J. Immunol	51	1.31	3794	5.422
Dig. Dis. Sci	45	1.16	1252	3.199
Proc. Natl. Acad. Sci. U. S. A	45	1.16	13289	11.205
Front. Immunol	44	1.13	981	7.561
Am. J. Physiol.-Gastroint. Liver Physiol	43	1.11	2692	4.052
Curr. Opin. Gastroenterol	40	1.03	1767	3.287
Nutrients	39	1.00	604	5.717
J. Clin. Gastroenterol	37	0.95	1626	3.062
Mucosal Immunol	36	0.93	1550	7.313
J. Pediatr. Gastroenterol. Nutr	32	0.82	992	2.839

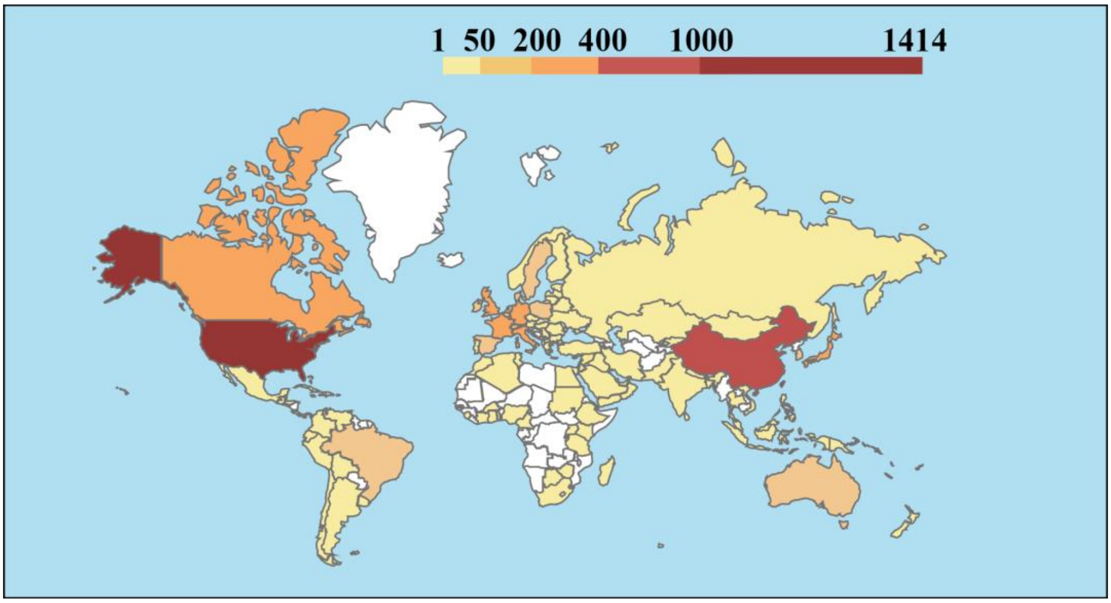

Figure S1. The global distribution of published articles from 2000 to 2020.

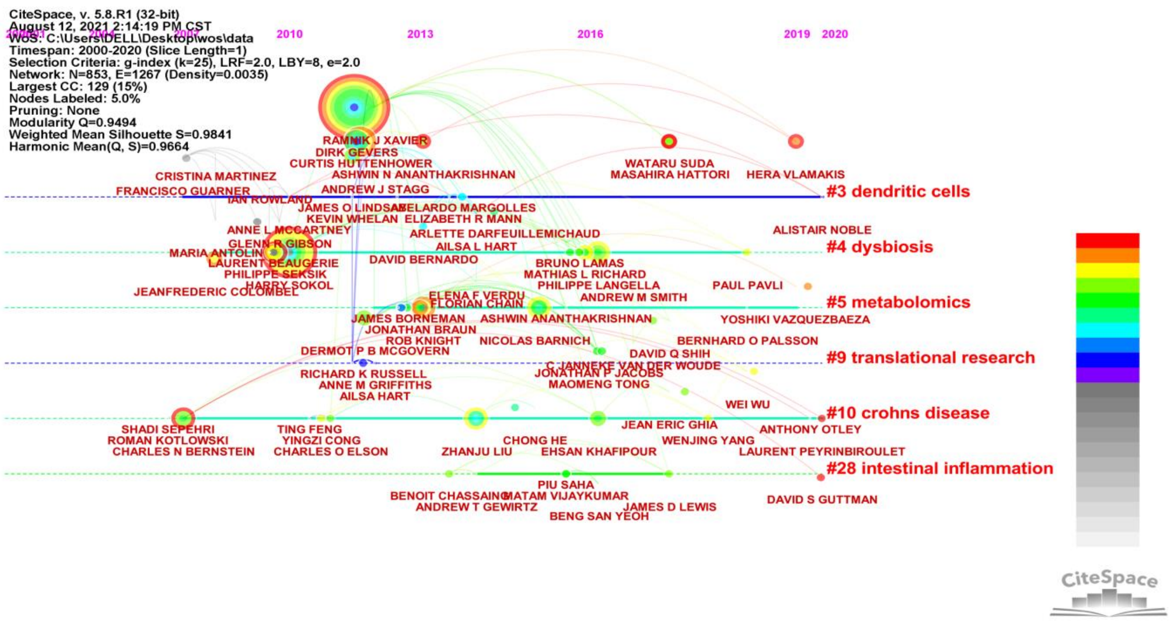

Figure S2. Timeline of author clusters. The timeline runs from left to right. The clusters are displayed from top to bottom in descending order of their size. The earliest cluster burst point was formed in 2002 and appeared in cluster #10. Around 2010, the main focus shifted to cluster #4, and after 2012, shifted to cluster #3.

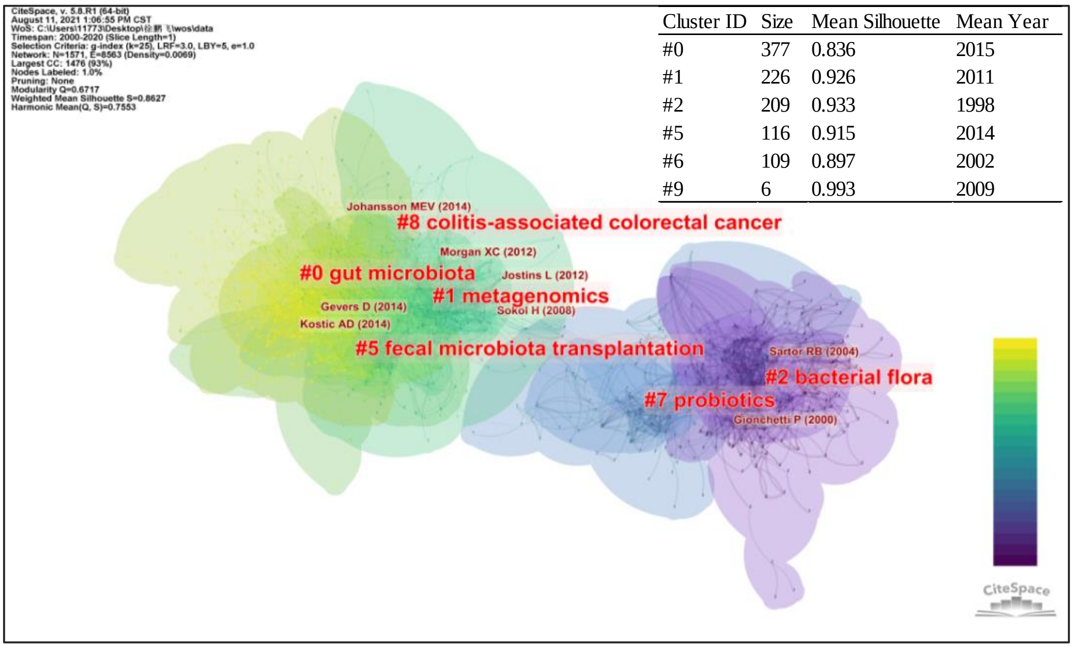

Cluster ID	Size	Mean Silhouette	Mean Year
#0	377	0.836	2015
#1	226	0.926	2011
#2	209	0.933	1998
#5	116	0.915	2014
#6	109	0.897	2002
#9	6	0.993	2009

Figure S3. Cluster visualization of the co-cited network of references via Citespace, together with the details and the most representative references of the generated clusters.

Cluster	References	Year	Strength	Begin	End	2000 - 2020
#0	Gevers D, 2014, CELL HOST MICROBE, V15, P382, DOI 10.1016/j.chom.2014.02.005, DOI	2014	**66.63**	2016	2020	
#1	Sokol H, 2008, P NATL ACAD SCI USA, V105, P16731, DOI 10.1073/pnas.0804812105, DOI	2008	**60.74**	2009	2013	
#7	Frank DN, 2007, P NATL ACAD SCI USA, V104, P13780, DOI 10.1073/pnas.0706625104, DOI	2007	**58.58**	2008	2012	
#0	Swidsinski A, 2002, GASTROENTEROLOGY, V122, P44, DOI 10.1053/gast.2002.30294, DOI	2002	**49.27**	2002	2007	
#8	Morgan XC, 2012, GENOME BIOL, V13, P0, DOI 10.1186/gb-2012-13-9-r79, DOI	2012	**48.22**	2014	2017	
#1	Jostins L, 2012, NATURE, V491, P119, DOI 10.1038/nature11582, DOI	2012	**47.49**	2013	2017	
#2	Sartor RB, 2008, GASTROENTEROLOGY, V134, P577, DOI 10.1053/j.gastro.2007.11.059, DOI	2008	**46.2**	2008	2013	
#5	Kostic AD, 2014, GASTROENTEROLOGY, V146, P1489, DOI 10.1053/j.gastro.2014.02.009, DOI	2014	**45.34**	2016	2020	

Figure S4. Top 8 references with the strongest citation bursts. The green bar represents the time period when the reference is cited and the red bar represents the time period when the cited reference appeared as a burst.
